# Claiming Food Ethics as a Pillar of Food Security †

**DOI:** 10.3390/foods15020255

**Published:** 2026-01-10

**Authors:** Ioana Mihaela Balan, Teodor Ioan Trasca, Nicoleta Mateoc-Sirb, Bogdan Petru Radoi, Ciprian Ioan Rujescu, Monica Ocnean, Flaviu Bob, Liviu Athos Tamas, Adrian Daniel Gencia, Alexandru Jadaneant

**Affiliations:** 1 University of Life Sciences “King Mihai I” from Timisoara, 300645 Timisoara, Romania; ioanabalan@usvt.ro (I.M.B.); rujescu@usvt.ro (C.I.R.); monicaocnean@usvt.ro (M.O.); 2 University of Agronomic Sciences and Veterinary Medicine of Bucharest, 011464 Bucharest, Romania; 3 Research Center for Sustainable Rural Development of Romania, Timisoara Branch, Romanian Academy, 010071 Bucharest, Romania; 4 Department of Internal Medicine II-Nephrology University Clinic, “Victor Babes” University of Medicine and Pharmacy, 300041 Timisoara, Romania; bob.flaviu@umft.ro; 5 Department of Biochemistry and Pharmacology, “Victor Babes” University of Medicine and Pharmacy, 300041 Timisoara, Romania; tamas.liviu@umft.ro; 6 West University of Timisoara, 300223 Timisoara, Romania; adrian.gencia@e-uvt.ro (A.D.G.); alexandru.jadaneant@e-uvt.ro (A.J.)

**Keywords:** food citizenship, food waste, ethical consumption, social equity, moral responsibility, FAO pillars, sustainable food systems, public policy, SDGs

## Abstract

This article explores the integration of food ethics as a proposed fifth and emerging pillar of food security, complementing the four dimensions established by the FAO 1996 framework (availability, accessibility, utilization, and stability). Using Romania as a case study, the research combines descriptive statistical analysis, legislative review, and conceptual interpretation to examine how moral responsibility, social equity, and food citizenship shape sustainable food systems. Quantitative data from Eurostat (2020–2022) reveal that Romania generates over 3.4 million tons of food waste annually, with households accounting for more than half of the total. This wasted abundance coexists with persistent food insecurity, affecting 14.7% of the population who cannot afford a protein-based meal even once every second day. Given the short time series (*n* = 3), including the entire data that was reported to date and the exclusive use of secondary data, the statistical results are interpreted descriptively and, where applicable, exploratorily. In this context, the findings demonstrate that food waste is not merely an issue of economic inefficiency, but rather a profound ethical and social imbalance. This research argues for the conceptual recognition of an ethical pillar within the food security framework linking moral awareness, responsible consumption, and equitable access to food. By advancing food ethics as a normative and societal foundation of sustainable food systems, this article offers a framework relevant for policy design, civic engagement, and collective responsibility, reframing food security beyond a purely technical objective.

## 1. Introduction

This research was originally presented at the 6th International Electronic Conference on Foods—Future Horizons in Foods and Sustainability, Part of the International Electronic Conference on Foods series, 28–30 October 2025, with the abstract being published as part of the conference materials [1].

In recent decades, food security has become one of the most discussed topics on the global agenda in the context of systemic challenges generated by climate change, economic inequalities, armed conflicts, and supply chain instability. The classic definition of food security, adopted by the Food and Agriculture Organization of the United Nations (FAO) in 1996, is based on four essential pillars: the availability of food, physical and economic access to it, its use in safe and nutritious conditions, and the stability of these factors over time [2]. This conceptual framework has provided a robust foundation for national and international policies aimed at combating hunger, improving nutrition, and strengthening the resilience of agri-food systems.

However, emerging evidence suggests the four pillars may be insufficient to fully address the ethical and social contradictions of contemporary food systems, particularly in contexts where food waste coexists with persistent food insecurity [3]. Especially in the European space, where the coexistence of excessive food waste and persistent food insecurity calls into question the equity of the food system, a reconfiguration of the theoretical and normative framework becomes necessary. According to data from the European Commission, approximately 20% of all food produced annually in the European Union is lost or wasted, while over 42 million citizens cannot afford a healthy meal every day [4]. This coexistence between excess and lack is not just a statistical imbalance, but reflects a deep moral fracture in food governance that cannot be remedied solely by technical or economic solutions. While existing policies within the four pillars can mitigate parts of this imbalance through redistribution, affordability measures, and waste-prevention instruments, they do not directly address the underlying normative contradiction between surplus and deprivation, hence the need for an explicit ethical dimension rather than policy adjustment alone.

The literature increasingly highlights the limitations of the current food security framework, particularly the absence of explicit considerations regarding moral responsibility, social equity, and ethical sustainability of food use [5,6]. Building on these strands of the literature, the present article advances food ethics as an explicit, emerging pillar of food security, conceptually extending the FAO framework [2]. This concept involves the inclusion of principles of moral responsibility, social equity, and civic engagement on the entire food chain from production to distribution, consumption, and waste. Initiatives such as the EAT-Lancet report [7], the IPES-Food contributions [8], as well as the FAO debates on the right to food [9], are already shaping a conceptual space in which food security is no longer analyzed exclusively in terms of quantitative access to food, but also in terms of distributive justice, responsibility towards resources, and the way in which consumers relate ethically to food. Against this conceptual backdrop, our article proposes the conceptualization of an “ethical pillar” of food security as an extension of the FAO (1996) framework [2]. This new dimension integrates values of equity, responsibility, and food citizenship, offering a perspective that complements the traditional approach focused on availability, access, utilization, and stability.

Romania is a revealing case study in this regard. Romania simultaneously faces high levels of food waste and a significant degree of food insecurity among vulnerable populations. According to the Ministry of Agriculture and Rural Development, Romania generates over 2.2 million tons of food waste annually, of which approximately 70% comes from households, retail, and the HoReCa sector [10]. At the same time, Eurostat data shows that 14.7% of Romanians cannot afford to consume a meal with meat, chicken, fish (or vegetarian equivalent) every second day [11]. This conjunction between excess and lack, between wasted abundance and daily shortages, expresses an ethical incongruity that cannot be ignored in the formulation of national food policies.

Despite these realities, the Romanian legislative framework is beginning to reflect an increased concern for the institutionalization of responsibility in the food sector. Law no. 217/2016 on reducing food waste, recently amended, imposes obligations on economic operators in the agri-food chain regarding the prevention, donation, and reuse of food [12]. At the same time, national platforms such as “We Reduce Waste” (orig. “Reducem Risipa”) promote responsible consumption behaviours and initiatives to redistribute food surplus. However, legislative and institutional changes are not enough if they are not accompanied by a transformation of individual and collective values. Sociological studies show that only 12% of Romanian consumers take ethical aspects into account when deciding to throw away food [13], which indicates a major gap between regulation and social awareness.

For this reason, our article argues for the inclusion of a new dimension in the analysis of food security: the “ethical pillar”, centred on the concept of food ethics and the mobilization of food citizenship as an active form of individual involvement in shaping a just, sustainable, and inclusive food system. The concept of food citizenship refers to citizens assuming democratic responsibility for the food system through informed consumption choices, combating waste, and supporting fair and sustainable practices [14].

This perspective aims to go beyond the predominantly technocratic approach to food security policies and highlight the fact that individual morality and collective responsibility must play a central role in rebuilding food systems. Thus, the main objective of this article is to argue that a sustainable food security cannot be achieved without a deep ethical anchoring, which not only includes the right to have food, but also a broader ethical responsibility governing food-related decisions across production, distribution, consumption, and waste. The present article therefore has a double aim: on the one hand, it provides a critical analysis of the current food security framework based on the Romania case study using relevant statistical and legislative data; on the other hand, it proposes a conceptual extension of this framework by integrating the ethical dimension in an effort to redefine food security as a collective right, shared responsibility, and expression of social justice. Specifically, this conceptual and policy analysis article proposes the conceptualization of an “ethical pillar” of food security as an extension of the FAO framework, integrating dimensions such as equity, responsibility, and food citizenship.

This article makes an original contribution to the specialized literature by advancing the idea of a “*food ethics pillar*” and offers a set of policy-relevant recommendations on how this dimension can be operationalized in public policies, educational programmemes, and everyday behaviours. Romania thus becomes a relevant example for other countries at the intersection of strong food traditions, socio-economic challenges, and emerging legislative transitions, not only through its national specificity, but also as a representative of a reality common to several states in transition between emerging regulations and food behaviours deeply anchored in traditions.

Against this background, the article addresses the following research questions:

Can food ethics be operationalized as a measurable dimension of food security?
To what extent do behavioural factors mediate the relationship between food waste and food insecurity in the Romanian context?

This paper is organized as follows: Section 2 presents the conceptual framework, Section 3 describes the data sources and methodological approach, Section 4 reports the results, Section 5 discusses the findings and policy implications, and Section 6 concludes the paper.

## 2. Conceptual Framework

### 2.1. The Ethical Dimension of Food Security—What Does Food Ethics Mean?

The concept of food ethics refers to the set of moral principles that should guide practices related to the production, distribution, consumption, and waste of food. It is a perspective that goes beyond economic or technical logic and invites reflection on equity in access to food, responsibility towards natural resources, the dignity of those involved in the food chain, and the long-term effects on human health and ecosystems.

Essentially, food ethics raises questions such as *Who has access to quality food? Who is excluded? What does “fairness” mean in the allocation of food? Is it moral to throw away food while others go hungry?* This approach brings together dimensions that have so far been treated separately, food security, environmental sustainability, public health, and human rights, without replacing existing frameworks such as food justice or food sovereignty [15].

The EAT-Lancet report explicitly states that food systems must be transformed to become “healthy for people and sustainable for the planet”—a formulation that implies a collective moral commitment [16]. Similarly, the contemporary academic literature insists that food should not be viewed simply as an economic good, but rather as a common good with profound cultural, social, and moral valences [17,18].

Integrating the ethical dimension into food security therefore not only means improving access, but also evaluating how this access is provided, at what social and ecological costs, and to what extent it is morally just.

While closely related to concepts such as food justice and food sovereignty, food ethics is analytically distinct in its primary focus. Food justice emphasizes equity, power relations, and rights within food systems, while food sovereignty prioritizes political autonomy, control over food systems, and the rights of producers and communities [15,16]. In contrast, food ethics operates as a normative framework that addresses moral responsibility across the entire food chain, including production, distribution, consumption, and waste, focusing on individual and collective ethical agency rather than institutional or political claims alone.

### 2.2. Food Citizenship—Democratic Responsibility and Participation

Food citizenship is an emerging concept that reflects the idea that individuals are not just passive consumers, but active actors who can positively influence the food system through conscious choices, local activism, and civic engagement. The term was first formulated in the works of Wilkins in 2005 [19], then developed by Carolan in 2011 [20] and Renting et al. in 2012 [14], and was gradually adopted within the FAO and IPES-Food programmes as a key tool for food democratization [21,22].

Food citizenship involves assuming ethical responsibilities in relation to everyday choices: preferring local and seasonal products, reducing waste, supporting fair and sustainable food systems, and getting involved in community initiatives for urban gardening, food education, or food redistribution.

It is a form of active citizenship that transcends the formal political sphere and is expressed through informed and supportive food behaviours. Moreover, food citizenship involves awareness of global interdependencies and the fact that the way we eat influences not only our personal health, but also the rights of others and the balance of ecosystems [14].

In the Romanian context, where food behaviours are deeply marked by traditions and also by the influences of modern consumerism, promoting food citizenship becomes essential for an authentic transition towards sustainability and food justice, while acknowledging structural and market constraints shaping consumer choices. It can build on the strong cultural traditions of home cooking, seasonal preservation (e.g., canning and pickling), and informal food-sharing practices within extended families and local communities. These practices can be reframed as civic agency by encouraging informed consumption choices, reduced food waste, and the solidarity-based redistribution of edible surpluses.

### 2.3. Ethics, the Right to Food, and Collective Responsibility

The right to food is recognized as a fundamental human right in numerous international documents, including the Universal Declaration of Human Rights (art. 25) and the International Covenant on Economic, Social and Cultural Rights (art. 11) [23,24]. According to the FAO, this right not only implies the absence of hunger, but also constant physical and economic access to nutritionally adequate, culturally acceptable, and sustainably obtained food [21].

But the right to food cannot be guaranteed without a collective dimension of responsibility—governmental, community, and individual. Food ethics adds a normative component to this right: it is not enough to “provide” food, it is necessary for the whole of society to participate in a system that does not exclude, waste, or degrade resources, alongside institutional and policy-based mechanisms.

Collective responsibility means assuming mechanisms for redistributing food surpluses, supporting vulnerable people, and preventing food losses through effective public policies and civic education. In this sense, the concept of food citizenship becomes the link between the ethics of the system and the responsibility of the actors that compose it.

### 2.4. The Recent Literature and Foundations for a New Framework

Over the past decade, a growing body of scientific literature has called for a reassessment of the framework for analyzing food security. IPES-Food warns that predominantly technocratic approaches ignore the social and moral dimensions of the global food crisis. The report *The Politics of Protein* draws attention to the fact that proposed technological solutions (e.g., cultured meat, protein supplements) may perpetuate inequalities if not accompanied by ethical and democratic reforms [22].

The FAO, through its annual SOFI reports, is gradually integrating components such as resilience, agency, and sustainability, but without formalizing them into a distinct pillar [25]. EAT-Lancet Commission report was among the most influential recent report, which called for the transformation of food systems “through the lens of equity and sustainability” as a precondition for achieving SDG2: Zero Hunger [7].

However, despite these contributions, there is still no clear explicit recognition of an “ethical pillar” within the FAO framework. One reason an explicit ethical pillar has not been formalized within the FAO framework is methodological: ethical dimensions are inherently more difficult to translate into standardized, comparable indicators than the four established pillars, which are anchored in measurable availability, access, utilization, and stability metrics. In addition, institutional frameworks tend to prioritize operational categories that can be monitored consistently across countries, while normative dimensions often remain implicit or dispersed across policy domains. Finally, the formal recognition of ethics as a pillar would require broad international consensus on normative content and measurement approaches, which remains challenging given cultural diversity and differing policy priorities. This is where the contribution of the present article comes in: proposing an integrated framework that explicitly includes the ethical dimension and food citizenship in the conceptual architecture of food security.

### 2.5. Theoretical Justification for a Fifth Pillar

Ethical considerations are often treated as cross-cutting elements within the existing FAO framework of food security, implicitly embedded in issues of access, utilization, and stability [2,5,6]. While this integrative approach acknowledges the presence of ethical concerns, it remains analytically insufficient. Cross-cutting integration tends to diffuse ethical responsibility across multiple dimensions, reducing it to an implicit background condition rather than a distinct object of analysis and governance.

A pillar-level status is theoretically justified when a dimension demonstrates normative distinctiveness, system-wide relevance, and analytical autonomy. Food ethics meets these conditions by introducing a normative layer that is not reducible to availability, access, utilization, or stability, but instead shapes moral orientation and responsibility across the entire food chain, including production, distribution, consumption, and waste [7,8,9]. Unlike purely transversal considerations, an explicit pillar enables ethical responsibility to be examined systematically and integrated into policy design, civic engagement, and evaluative frameworks [14].

Moreover, treating food ethics solely as transversal principal risks perpetuating the gap between technical policy instruments and actual food-related behaviours. Pillar-level recognition makes ethical considerations visible and conceptually central, supporting future efforts to develop dedicated indicators and governance mechanisms aligned with contemporary food-system contradictions, including the coexistence of food waste and food insecurity [2,5,6].

## 3. Materials and Methods

This research was built on a combination of descriptive quantitative analysis, statistical analysis, legislative review, and conceptual interpretation, primarily descriptive and exploratory, aiming to integrate the ethical dimension into the food security framework. In particular, this study focuses on Romania using official secondary data, complemented by interpretive analysis of the normative and behavioural context. This research is part of a research-for-policy approach, not only aiming to understand current food phenomena, but also to support the development of more equitable, ethical, and sustainable public policies in the field of food security.

Romania was chosen as a case study because it offers a complex and revealing analytical terrain for exploring the ethical dimension of food security, and because all authors are institutionally based in Romania, allowing for direct familiarity with the national context [26,27,28]. On the one hand, the country faces significant challenges related to food insecurity among the vulnerable population, despite being an agrarian state with high production potential [29,30]. On the other hand, data on food waste in the household sector and the HoReCa industry indicate a level of consumption that is unbalanced and often disconnected from responsibility towards resources [31]. This coexistence between abundance and deprivation, between wasted surplus and daily shortages, makes Romania a paradigmatic example for analyzing the ethical paradoxes in modern food systems.

Moreover, Romania is at the intersection of Eastern European food traditions and the pressures of European convergence, being simultaneously subject to both local cultural legacies (such as the habit of throwing a little on the plate out of “social respect”) and European food policies oriented towards efficiency, waste reduction, and inclusion. Thus, Romanian food behaviours not only reflect a national reality, but also global tensions between consumer individualism and collective responsibility.

This positioning makes Romania function as a microcosm of global food contradictions, especially in transition or middle-developed countries, where economic growth indicators coexist with persistent inequalities and aspirational consumption patterns. Therefore, this study’s results may be relevant and transferable to other international contexts, especially for countries in Central and Eastern Europe, Central Asia, or Latin America, where similar configurations of insufficient access, waste, and responsibility in the food sector are found.

### 3.1. Data Sources

The most recent statistical data from official and international sources were used to ensure coherence and comparability at the European level:

• FAOSTAT for general indicators on food security and food losses [32];
• Eurostat for indicators on the population’s access to nutritious food, including data on food deprivation, access to animal protein, income, and inequalities [33,34];
• Ministry of Agriculture and Rural Development and National Sanitary, Veterinary and Food Safety Authority (ANSVSA) for specific data regarding the volume of food waste, sources of generation, and control measures [10,28,31,35];
• National legislation, in particular, Law No. 217/2016 on reducing food waste (updated version in 2024) [12];
• Specialized literature for conceptual analysis of the ethical framework.

All data collected refer to the period 2020–2022, representing the most recent figures reported at the time of analysis, ensuring the contemporary relevance of the results. The analysis focused on national indicators for Romania, with occasional references to the European Union average for contextual comparison.

The selected time frame (2020–2022) requires contextual clarification. The years 2020 and 2021 coincide with the COVID-19 pandemic, which temporarily altered food-related behaviours, including consumption patterns, food purchasing, and waste generation, due to lockdowns, mobility restrictions, and disruptions in food services. These conditions may have influenced behavioural responses, particularly at the household level, and therefore limit the direct generalization of results to non-crisis periods.

We reiterate the aspect that, at the time of data extraction for this study, 2022 represented the most recent year reported in the consulted sources for the indicators included in the analysis. Consequently, the selected period reflects a data availability constraint rather than an analytical preference, and this temporal limitation is acknowledged as affecting the generalizability of the findings beyond the observed interval.

### 3.2. Analytical Methodology

The methodology involved several stages:

• *Descriptive analysis of statistical data*. Relevant indicators for the four pillars of food security (availability, accessibility, utilization, stability) were selected and correlated with data on food waste and food insecurity. In particular, the following were analyzed:

- The amount of food waste generated annually in Romania;
- The structure of generation sources (households, retail, HoReCa);
- The share of the population that cannot afford a regular nutritious meal;
- The proportion of consumers who consider ethical aspects in their eating behaviour.

• *Regulatory and legislative analysis.* The evolution of the legal framework regarding food waste prevention, the obligation of donation/reuse, and the incentives offered for responsible food behaviour were analyzed. The extent to which these regulations institutionalize ethical responsibility was also evaluated.
• *Interpretive and conceptual analysis.* The data were interpreted in relation to the conceptual assumption of the paper, according to which the current food security framework is insufficient without the integration of an ethical dimension. The correlation between statistics, norms, and behaviours was thus traced to highlight the need to conceptually articulate a fifth pillar: the ethical one.
• *Graphic illustration and visual synthesis.* Tables and figures were produced that summarize the relationship between food ethics, food waste, and food security, highlighting the expansion of the FAO framework by including the food ethics pillar.

### 3.3. Statistical Methodology

A statistical analysis was carried out to assess the relationships in an exploratory manner between the food chain segments that contribute to the generation of food waste in Romania and to quantify their impact on the total volume of losses. The PATH analysis was employed exclusively as an exploratory, model-implied, and scenario-based analytical tool, aimed at illustrating potential interdependencies between food-chain segments under conditions of very limited data availability. The analysis was not intended as a confirmatory structural equation model, and therefore it was not evaluated using standard goodness-of-fit indices (CFI, RMSEA, SRMR). The resulting PATH diagrams should be interpreted as illustrative representations of hypothesized directional relationships, rather than as empirically validated structural models.

The dataset used comes from the Eurostat database—Food Waste Data Collection (2020–2022, respectively, the latest reported data), which reports data expressed in total tons, in accordance with the official methodology of the European Union. The analysis targeted the five main segments:

• Total (aggregate changing according to the context)—T.
• Primary production of food—agriculture, fishing, and aquaculture—PPF.
• Manufacture of food products and beverages—MFP.
• Retail and other distribution of food—RDF.
• Restaurants and food services—RFS.
• Total activities by households—TAH.

#### 3.3.1. Analysis of the Percentage Contribution of Variables to Total Waste

To calculate the contribution of each segment of the food chain to total waste, the following relationship was used:

(1) $$C_{i}=\frac{\left|∆x_{i}(t)\right|}{\sum_{i}\left|∆x_{i}(t)\right|}\times 100\;$$

where

*C_i_* [%]—the contribution of the food chain segment “i” to total waste;

*t*—data reporting year;

$x_{i}\left(t\right)$—the value of waste for segment, “*i*” in reporting year “*t*”;

$∆x_{i}\left(t\right)=x_{i}\left(t\right)-x_{i}\left(t-1\right)$, variation of waste compared to the previous year, for segment “*i*”.

By ranking the values *C_i_*, an image of the contribution of each segment of the food chain to the variation in total waste will be obtained.

#### 3.3.2. Correlation Analysis of Each Segment of the Food Chain with Total Waste

The linear correlation coefficient between the annual series of each food chain segment and the annual series of total waste was calculated. This assesses the intensity of the link that each food chain segment has with total waste.

The expression for Pearson’s linear correlation coefficient “*r_i_*” for two statistical data series, $x_{i}\left(t\right)$ and $y_{i}\left(t\right)$, is [36]

(2) $$r_{i}=\frac{\sum_{t}\left(x_{i}\left(t\right)-{\bar{x}}_{i}\right)\left(y_{i}\left(t\right)-{\bar{y}}_{i}\right)}{\sqrt{\sum_{t}{\left(x_{i}\left(t\right)-{\bar{x}}_{i}\right)}^{2}}\sqrt{\sum_{t}{\left(y_{i}\left(t\right)-{\bar{y}}_{i}\right)}^{2}}}$$

where

*r*_i_—the linear correlation coefficient between the multiannual series $x_{i}\left(t\right)\;$ of each food chain segment “*i*” and the multiannual series of total waste $y_{i}\left(t\right)$;

${\bar{x}}_{i},\;{\bar{y}}_{i}$—the average values of the two series, $x_{i}\left(t\right)$ and $y_{i}\left(t\right)$.

The interpretation of the “*r_k_*” values is as follows: “*r*” belongs to the interval [−1, 1] (r∈[−1, 1]). “*r*” values close to 1 show that the series have the same trend, increasing or decreasing together, so there is a direct correlation. Values close to –1 indicate a reverse trend of evolution, hence an inverse correlation. Values close to 0 indicate the absence of correlation. Given the limited number of observations, correlation coefficients are interpreted descriptively and not in an inferential statistical sense.

#### 3.3.3. PATH Analysis

Because the RFS and TAH segments predominantly reflect individual decision-making and consumer behaviour, the variations observed in these areas cannot be explained solely by isolated economic or structural factors. Food waste in these food chain segments results primarily from everyday practices (such as purchasing habits, portioning, storage, and waste management) that are shaped by cultural norms and value perceptions, rather than technological limitations. For this reason, a PATH analysis was employed as an exploratory analytical tool to identify how changes occurring within the RFS and TAH propagate through the broader food system and influence overall levels of food waste, highlighting the behavioural interdependencies embedded throughout the chain.

Given the short time series available, since the statistical data on food waste in Romania are carried out over a period of 3 years, a short period of time, according to the currently existing FAO records (2020–2022), the annual series for the 3 years were the basis for a linear extension to construct a short-term scenario for a period of another three years.

This extension was achieved by keeping the slope value of the linear regression function determined by the values reported in the three years constant. Even if the real evolution trend may be any, for short time segments, the linear function can also approximate other functional models. Thus, we created a short-term evolution scenario that allows us to identify at an exploratory level, links between variables. This evolution scenario has a high probability of fulfilment because consumer eating behaviour changes slowly over time. The low number of data reported so far does not yet allow us, at this point in time, to establish and statistically confirm the values of the obtained parameters. However, even with these limitations, the models developed for exploratory purposes can allow us to make preliminary observations on how the RFS and TAH segments have potential implications on food waste in the other evaluated segments. This linear extension applied in this study does not aim to model behavioural processes or imply linear behavioural dynamics. Instead, it serves as a minimal, short-term scenario-building device used solely for exploratory illustration of potential interdependencies under conditions of very limited data availability, relying on short-term behavioural inertia rather than assumptions of behavioural linearity.

The trends thus obtained represented the inputs of the PATH analysis. Thus, the extent to which food waste in restaurants and households may be associated with food waste in each segment of the chain was explored. The JASP 0.17.3.0 software application was used with the Structural Equation Modelling procedure. The spreadsheet calculations were performed using Microsoft Excel 365 [37,38]. This scenario-based extension does not aim to predict future values, but rather to enable preliminary observation of potential interdependencies between food chain segments. The PATH analysis should be interpreted as a model-implied exploration of potential inter-segment relationships, rather than as a representation of underlying behavioural mechanisms.

All Eurostat food waste values used in this study are national totals for Romania reported in tonnes per year (t/year) for 2020–2022. Per capita values (kg/capita) were derived by dividing annual totals by the resident population and converting tonnes to kilograms. Percentage contributions (Ci) are reported in %. Correlation coefficients (r) and coefficients of determination (R^2^) are unitless. In the PATH/SEM outputs, regression coefficients (β) and residual covariances are reported as the unitless quantities provided by the software within the specified model structure. The Eurostat Romania series for 2020–2022 contained no missing values; therefore, no imputations were performed.

### 3.4. Ethical Considerations

This research was based exclusively on secondary data, coming from official, public, and accessible sources, such as international databases (FAOSTAT, Eurostat), government reports, and legislative documents. Therefore, it did not involve the collection of personal data, the performance of experiments on human subjects, or the use of methods that would require prior approval by an ethics committee.

At the same time, at all stages of the research, the fundamental principles of scientific integrity were respected, including

• The transparency of sources and correct attribution of ideas and data used;
• The objectivity of analyses and the avoidance of drawing conclusions that are not supported by the data;
• Respect for human dignity through emphasis on social responsibility in food security;
• Commitment to equity, reflected in the proposal of a conceptual framework that integrates food ethics and citizenship as essential elements of public policies.

Also, in interpreting the phenomenon of food waste and insecurity, we avoided stigmatizing vulnerable groups and sought to highlight the structural and systemic causes that generate such imbalances.

## 4. Results

### 4.1. Quantitative Assessment of Food Waste and Food Insecurity

Between 2020 and 2022, Romania generated between 3.2 and 3.45 million tonnes of food waste annually, according to the latest data published by Eurostat, and this indicates an increasing trend over the observed period. Relative to the population, this volume corresponds to an increase from 166 kg/person in 2020 to 181 kg/person in 2022, which places Romania slightly below the European Union average (around 185 kg/person), but indicates a more accelerated growth than in other Member States with similar levels of development [11].

Detailed data analysis (Table 1) highlights an unbalanced distribution of food losses along the agri-food chain, reported at the level of the entire population. Households remain the main source of waste, generating between 52% and 55% of the total, with an increase from 1.66 million tonnes in 2020 to 1.88 million tonnes in 2022. This upward trend suggests the limited effectiveness of national awareness and prevention programmes, despite the implementation of Law no. 217/2016 on the reduction in food waste (revised in 2023). The HoReCa sector (restaurants, public food services, catering) ranks second, with values between 0.48 and 0.59 million tonnes/year, followed by primary production and the food industry, which together account for approximately one third of total waste. The relatively high share of restaurants and food services (HoReCa) in Romania (2020–2022) may be associated with sector-specific practices such as demand uncertainty, overproduction to avoid stockouts, portion sizing and menu complexity, and limited uptake of systematic prevention and safe redistribution mechanisms. This pattern should also be interpreted contextually, as the sectoral ordering can differ across EU Member States due to structural differences in food systems and, in some cases, differences in reporting and aggregation practices; therefore, cross-country comparisons are discussed descriptively. These sectors are associated with vulnerabilities in post-harvest management, storage, and processing, especially in the lack of efficient logistics infrastructures.

Although the total increase of approximately 7.8% over the three years seems moderate from a statistical perspective, it has relevant ethical implications. Every ton of wasted food not only represents a loss of resources, labour, and energy, but also social inequity, ethical, and social concerns in a society where over 14% of citizens cannot afford a protein meal at least once every two days [40,41]. The coexistence of these two realities—wasted abundance and food deprivation—illustrates one of the most visible moral paradoxes of the contemporary Romanian food system.

The sectoral analysis highlights contrasting developments. While the processing industry recorded a sharp increase in food waste in 2022 (+40% compared to 2021), potentially because of the post-pandemic recovery and realignment of production flows, primary production, and retail remained relatively stable, suggesting more efficient inventory management. However, the continued dominance of household waste indicates the existence of a major gap between legislative regulation and effective social behaviours.

The per capita indicator confirms the same trend: household waste increased from 86 kg/person in 2020 to 99 kg/person in 2022, an increase of over 15% in just two years (Table 2).

**Figure 1:**
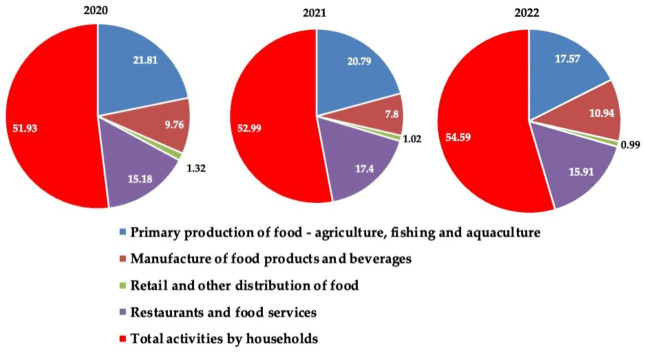
Percentage distribution of food waste across food chain segments in Romania (2020–2022). Source: Original by authors.

The restaurants and food services (HoReCa) sector also recorded an increase from 25 kg/person to 29 kg/person, in parallel with the relaunch of economic activities after the pandemic restrictions. These figures place Romania close to the European median, but still far from the “*Farm to Fork*” *Strategy* commitment to halve per capita food waste at retail and consumer levels by 2030. Figure 1 illustrates the distribution of food waste in Romania across the food chain segments.

From a food security perspective, these quantitative data indicate a double deficit. On the one hand, public policies focused on reducing food losses have not yet been internalized at the behavioural level, which may reflect a weak integration of the ethical dimension in the culture of consumption. On the other hand, the increase in waste coexists with the persistence of food insecurity, manifested by difficulties in accessing nutritious, diversified, and safe foods for significant segments of the population.

Therefore, the quantitative assessment indicates that food waste is not just a problem of economic efficiency, but also a problem of social and ethical justice. Every kilogram of food wasted affects the first two pillars of food security—availability and accessibility—reducing the resources available for equitable distribution and contradicting the principle of collective responsibility implied in the right to food.

### 4.2. Analysis of the Percentage Contribution of Food Chain Segments to the Variation in Total Waste

For all the PATH-based results presented in this section, coefficients are reported descriptively; *p*-values and standard errors are not reported, given the exploratory nature of the analysis and the very limited number of observations. The analysis of the variations in the period 2020–2021, presented in Table 3, allowed for the determination of the share of each sector of the food chain in the total food waste generated in Romania. To calculate them, the calculation relationship (1) was used. The RFS variable had an important share, over 35%, in the variation in total waste, and, together with TAH (47.27%), it cumulates over 80% of the variation in total waste. This fact shows that total food waste could be reduced by reducing it in the RFS segment. And in the period 2021–2022, a significant share of RFS in the variation in total waste was found, over 14%, and, cumulated with TAH (24.33%), it represents a significant share, even if it is lower than that of the previous year.

Given the short time series (*n* = 3), the contribution percentages reported in Table 3 should be interpreted descriptively rather than inferentially. The limited number of observations does not allow for the robust estimation of confidence intervals, and the reported values are intended to illustrate relative magnitudes rather than statistically bounded effects.

#### 4.2.1. Results of the Correlation Analysis of Each Segment of the Food Chain with Total Waste

By calculating the linear correlation coefficient, the intensity of the link between two data series is determined, and this fact indicates whether the annual series of each food chain segment and the annual series of total waste have a mathematical link. The data can be found in Table 4, along with the coefficient of determination R^2^. This coefficient indicates how much of the variation in total waste is explained by individual sectors through this model. In this context, it also becomes a qualitative indicator that describes the correlation of the analyzed variables. Due to the small number of observations (*n* = 3), correlation and determination coefficients are reported descriptively for the observed period and are not interpreted in an inferential framework.

The PPF sector indicates a medium-level correlation r = −0.69 with the annual series of total waste values, but an inverse correlation. Thus, we can say that, while PPF decreased in the period 2020–2022, the total waste increased. It is also the only inverse correlation observed between economic sectors with total food waste. In all other cases, the values of the correlation coefficients were positive. So, a trend of increasing waste at the level of a sector is also followed by an increase at the level of total waste.

Importantly, this inverse association should not be interpreted as evidence of an inverse causal effect. Given the very short time series (*n* = 3), correlation results are presented descriptively and may reflect broader structural or contextual shifts rather than a direct relationship between primary production waste and total waste. Plausible confounders include post-pandemic supply-chain adjustments (e.g., improvements in storage and distribution efficiency), changes in consumer demand and retail inventory practices, macroeconomic dynamics (e.g., GDP per capita changes and inflation affecting purchasing and waste behaviour), and policy or reporting changes during 2021–2022. These factors may alter sectoral waste levels in different directions over time. Future research using longer time series and multivariate designs is needed to test whether the observed pattern persists after accounting for such drivers.

A value r = 0.77 shows that the annual series of waste from RFS is strongly correlated with total waste, and, according to the coefficient of determination, 59% of the variation in total waste could be accounted for by the specified path from RFS within the model. The most important correlation is observed between TAH series and the annual series of total waste values, r = 0.99. A very significant share, 98%, of the total waste is associated with the TAH sector.

However, the way in which the food chain segments that predominantly involve individual decision-making—namely RFS and TAH—influence the final variation in total waste cannot be fully explained by descriptive analyses due to the complex interdependencies between economic sectors. Therefore, to highlight these causal relationships and synergistic effects between segments, a PATH analysis was further applied.

Given the short-observed time series (*n* = 3) and the exploratory scenario-based extension used for PATH modelling, no inferential statistics (*p*-values, standard errors) are reported and no statistical significance claims are made.

#### 4.2.2. Results of the PATH Analysis

PATH analysis was employed as an exploratory tool to complement the potentially subjective pictures that previously described in a singular way the extent to which a food chain segment is associated with the total waste. The interactions between variables can be explored using the PATH diagram below. In this exploratory, scenario-based specification, the term “causal variable” is used in a model-implied sense (i.e., directional paths specified a priori) and does not imply empirically validated causality. The regression coefficients (β) quantify the magnitude and direction of the specified paths within the model, indicating the expected change in an outcome associated with a one-unit change in the “causal” variable in this specification. Residual covariance values capture shared variation between variables that is not represented by the specified “causal” variable in the model. These quantities are dimensionless.

Because the PATH analysis is used here as an exploratory, model-implied, and scenario-based illustration rather than as a confirmatory structural equation model, standard goodness-of-fit indices (CFI, RMSEA, SRMR) are not reported by design. Accordingly, the PATH analysis is intended solely as an illustrative and exploratory visualization of model-implied interdependencies and should not be interpreted as empirical validation of structural or behavioural relationships.

The total number of pairs/groups of variables representing the interactions is very large. For this reason, this study only analyses the two most relevant models:

- RFS as a causal variable for the other variables.
- TAH as a causal variable for the other variables.

##### Model 1. RFS as a Causal Variable

**Figure 2:**
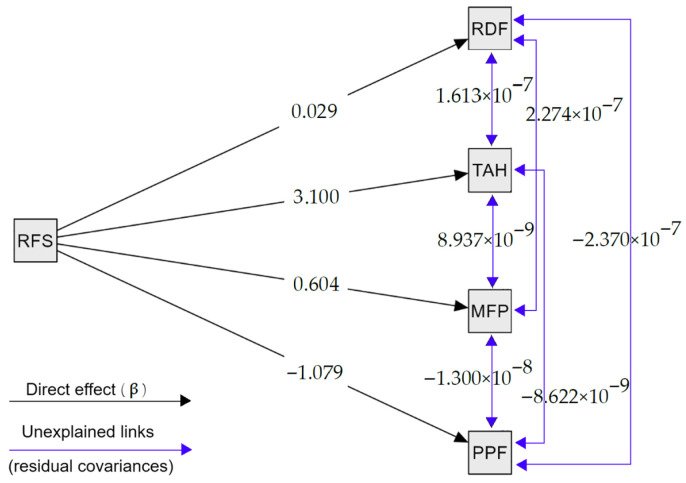
PATH diagram corresponding to Model 1 with RFS as the causal variable Source: Original by authors, processed using JASP 0.17.3.0 of Eurostat statistical data.

The first model only analyzes how waste in the RFS sector, as a causal variable, can induce changes in waste from other food chain segments, but also some interactions of these dependent food chain segments. Thus, the regression coefficients are indicated in Figure 2.

A change in the waste values in RFS is associated with a negligible change in food waste in the RDF sector, the regression coefficient being β = 0.03. The RFS sector also shows a direct link with the TAH sector regarding waste. The increase in waste in restaurants is also associated with a similar behaviour in households. The regression coefficient β = 3.1 indicates that, within the specified model, a 1 ton increase in RFS is associated with an estimated 3.1 ton increase in TAH. An opposite phenomenon was observed by the PPF sector. Waste from PPF level decreased, while waste from RFS increased. Thus, within the specified model, a 1 ton increase in RFS corresponds to is associated with an estimated decrease of 1.08 tons of waste in PPF, according to the regression coefficient β = −1.08. The coefficients of determination R2 indicate the proportion of the variation in waste in economic sectors that could be explained by the RFS. The values of the β and R^2^ coefficients are found in Table 5 [42,43,44,45,46].

However, there are also links between the dependent variables, PPF, MPF, RDF, and RFS, which are not explained by the causal variable in this context. These interactions are given by the residual covariance values in Table 6.

##### Model 2. TAH as a Causal Variable

**Figure 3:**
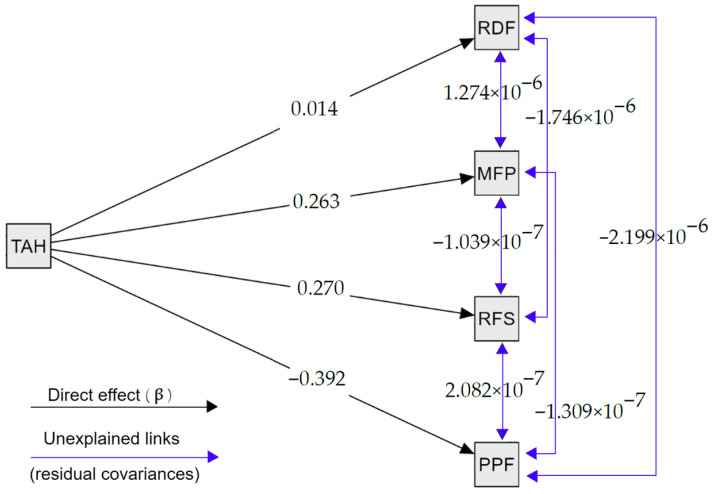
PATH diagram corresponding to Model 1 with TAH as causal variable. Source: Original by authors, processed using JASP 0.17.3.0 of Eurostat statistical data.

The second model only analyzes how waste in the TAH segment, as a causal variable, can induce changes in waste in other segments of the food chain and, respectively, other interactions. The PATH diagram is shown in Figure 3.

Given the limited number of observations, standard goodness-of-fit indices for structural models (e.g., CFI, RMSEA, SRMR) could not be reliably estimated. Accordingly, the PATH diagrams are presented as exploratory visual representations of model-implied relationships rather than as fully fitted structural models. The coefficients of model 2 are presented in Table 7 and Table 8.

In a similar way to the behaviour of RFS, a change in the waste values in TAH induces a negligible change in food waste in the RDF segment, the regression coefficient here being β = 0.01.

The TAH variable, like the RFS, also shows an inversion phenomenon. During the period under analysis, waste decreased in PPF while food waste increased in TAH. Thus, a one-ton increase in waste in TAH corresponds to a 0.39 ton decrease in PPF.

### 4.3. Legislative and Institutional Analysis—Integrating Ethics into National Food Policy

The quantitative results presented in the previous sections highlight a structural mismatch between the scale of food waste generation and the persistence of food insecurity, as well as the dominant contribution of households and downstream segments to total food waste. These empirical patterns point to the importance of governance mechanisms, regulatory frameworks, and institutional responsibility in shaping food-related practices. In this context, the following legislative analysis examines how the Romanian policy framework addresses food waste prevention, redistribution, and ethical responsibility and whether existing regulatory instruments are aligned with the observed empirical dynamics.

Romania’s legislative and institutional framework in the field of food safety and food waste has evolved visibly in the last decade, reflecting efforts to harmonize with European directives on the sustainability of food systems. However, although national legislation includes important normative instruments, the ethical dimension of food governance remains insufficiently outlined, being addressed rather indirectly through the general principles of social responsibility and environmental protection [34,39].

The adoption of Law No. 217/2016 on reducing food waste, subsequently amended by GEO No. 92/2021 and further clarified by HG No. 51/2019, represents a crucial step in the institutionalization of food responsibility [12,47,48]. The law provides explicit obligations for economic operators in the agri-food chain to prevent waste, redirect surplus to human consumption, and annually report the quantities of food recovered [9]. However, the implementation of the measures remains partial: available reports indicate that only a limited number of economic operators currently submit data under the national food waste reporting framework—donation centres, refrigerated transport, NGO networks—which is insufficiently developed [49].

In parallel, the *National Plan for Reducing Food Waste*” [50,51] establishes directions for action aligned with the European Commission’s “*Farm to Fork*” Strategy, which aims to reduce food losses by 50% by 2030 [52]. The document promotes four major axes—prevention, reuse, separate collection, and food education—but without including an explicit ethical dimension that is likely to transform waste reduction into a moral imperative and not just an economic or environmental obligation. The predominantly technical focus of these policies reflects a vision oriented towards efficiency, not equity.

Romania’s National Strategy for Sustainable Development 2030 [50] mentions food security as a strategic objective associated with SDG 1 (No Poverty), SDG 2 (Zero Hunger), and SDG 12 (Responsible Consumption and Production), insisting on “the need for a sustainable and responsible food culture”. However, the implementation of this vision has been limited to specific initiatives without the creation of a cross-sectoral framework for food ethics—although similar recommendations appear in FAO reports on the right to food [39] and in the IPES-Food analysis on governance ethics in food systems [8].

At the national institutional level, responsibilities are divided between several public entities:

• Ministry of Agriculture and Rural Development (MADR)—coordinator of policies on food loss reduction and redistribution;
• National Sanitary-Veterinary and Food Safety Authority (ANSVSA)—responsible for regulating food safety and food donations;
• Ministry of Environment, Waters and Forests (MMAP)—which integrates food waste into circular economy and waste management policies;
• Ministry of Education and Research (MEC)—limited involvement through educational programmes such as Green Week or waste prevention campaigns in schools [50].

This distribution of powers generates institutional fragmentation and coordination difficulties. Romania does not yet have an advisory council for food ethics like those in other European countries, for example, the Food Ethics Council in The United Kingdom or the Dutch Council for Animal Affairs (RDA), an independent expert council that advises the Minister of Agriculture on animal welfare and health in public policy [53,54].

In Romania, in the absence of such a body, an increasingly important role is played by civic initiatives. Platforms such as Food Waste Combat Association, “We Reduce Waste” (orig., *Reducem Risipa*”), “Food Bank” (orig., *Banca pentru Alimente*”), or ShareFood Romania act as mediators between producers, retailers, and vulnerable communities, actively contributing to the redistribution of food and changing behaviours [55,56,57]. These initiatives prefigure the emergence of a form of institutionalized food citizenship in which solidarity and moral responsibility become components of food governance. However, the lack of unified reporting mechanisms and a national database limits the integration of these efforts into public policies [10,58].

According to the press release of the Ministry of Agriculture and Rural Development, the recent amendments to Law no. 217/2016 on reducing food waste aimed to clarify the obligations of economic operators and introduce measures to stimulate food redistribution through partnerships between retailers, food banks, and NGOs. The new provisions also aim at a unified reporting of data at national level and the expansion of educational programmes on waste prevention, in line with the principles of the circular economy and the “*Farm to Fork*” Strategy [10,59].

According to the National Sanitary Veterinary and Food Safety Authority (ANSVSA), reducing food losses must be correlated with guaranteeing the safety of redistributed food by applying the related sanitary and veterinary norms, which gives this issue a double dimension: a technical one and an ethical one. ANSVSA emphasizes that “food that maintains its quality and safety until the expiration date should not be considered waste, but resources that can support people in difficulty”, a principle that fully reflects the spirit of the UN Agenda 2030 on reducing inequalities and promoting collective responsibility [35].

In the absence of a consolidated institutional culture of moral responsibility, transforming food behaviours at the individual level becomes the most promising space for operationalizing food ethics in Romanian society. By adopting an ethical framework that cuts across all sectors—from production to consumption—Romania can align more firmly with the UN Agenda 2030, in particular, SDG 12 on responsible consumption and production and SDG 2 on eradicating hunger. In this context, public policies must go beyond their purely normative role and become an instrument for cultivating civic and moral engagement with food and resources, strengthening the link between the right to food, resource sustainability, and social justice.

### 4.4. Behavioural and Cultural Insights—Ethical Awareness and Food Citizenship Among Romanian Consumers

Eating behaviours represent the most sensitive and, at the same time, the most ethically relevant dimension of food security. They not only reflect preferences and consumption habits, but also the level of moral awareness regarding the value of food, waste, and the impact of individual choices on the community. In Romania, transforming these behaviours remains one of the greatest challenges for the operationalization of food ethics and for the consolidation of an active form of food citizenship.

According to data provided by the European Commission through Flash Eurobarometer 520—Food Waste and Date Marking and Eurobarometer 535—Public Opinion in the European Union (Dataset: Food Waste and Responsible Consumption), 71% of Romanian citizens admit to throwing away food at least once a month while only 29% declare that they do not waste food at all [40,60,61]. The main causes identified are excessive food purchases (48%), lack of shopping planning (43%), and confusion related to the labels “to be consumed preference before” and “best before” (37%). These data confirm that waste is not the result of a lack of resources, but rather of everyday behaviours influenced by education, the perception of comfort, and the cultural relationship to food.

The results of recent studies conducted in Romania provide a detailed picture of these behaviours, namely, only 11.9% of survey respondents say that they take ethical or sustainability aspects into account when deciding to buy or throw away food, a percentage well below the European average (22%). At the same time, 68% declare themselves willing to donate surplus food, but only 8–9% do so, confirming the existence of a clear discrepancy between intention and action. Studies also show that adults under 25 from urban areas and with higher education show a greater concern about food waste, but the lack of adequate infrastructure for redistribution limits the transformation of ethical intentions into concrete behaviours [41,62].

Studies of the analyses of food behaviours and motivations associated with waste show that, in Romania, the perception of the value of food is deeply shaped by cultural factors [30,33,63]. In urban households, waste is often associated with the idea of “abundance” and high social status, while in rural areas conservative attitudes and traditional reuse practices (e.g., food waste transformation, composting, animal feeding) prevail [41,62,63]. This polarization between modernity and tradition reflects the tension between the culture of consumption and the culture of care for food, with direct implications for the implementation of waste reduction policies.

FAO data confirm that, in Europe and Central Asia, food education and the degree of nutritional literacy directly influence the level of food losses and ethical behaviours [9]. Countries that have introduced educational modules on food waste and sustainable consumption in school curricula have recorded significant reductions in waste. In Romania, such programmes are still limited, taking place sporadically through civic initiatives or school campaigns (e.g., Green Week, Food Waste Combat), without systematic integration into the national educational curriculum [52,55].

From a sociological perspective, food behaviours in Romania can be interpreted in the context of a dual food culture: a traditional one which values sobriety, agricultural work, and respect for food, and a modern one associated with urbanization, food industrialization, and aggressive marketing. This duality generates what we could call an “ethical fracture”: people know that waste is wrong, but continue to practice it out of cultural inertia, lack of time, or the false perception that food is an inexhaustible resource.

In practical terms, food citizenship can be operationalized through routine actions that directly reduce waste and support equity, such as planning purchases and portions, using date labels correctly, prioritizing leftover use and safe storage, redirecting edible surplus via food banks or local donation channels, choosing retailers and food services that adopt redistribution and transparency practices, and supporting local initiatives that shorten supply chains. These are small-scale behaviours, but they translate the ethical pillar into measurable everyday practices.

In this context, the concept of food citizenship offers a promising direction for reconnecting individual behaviours and collective responsibility. The food citizen is not just a rational consumer, but also a moral actor aware of the implications of their decisions. In Romania, incipient forms of food citizenship are manifested through participation in civic initiatives (food banks, donation programmes, local producers’ markets, consumer cooperatives), but these actions remain marginal compared to the magnitude of the waste phenomenon.

Against the backdrop of these developments, the cultural dimension of food ethics becomes essential. In the Romanian space, food has a symbolic and identity charge, being associated with hospitality, religiosity, and collective rituals. Therefore, any strategy for promoting food ethics must consider the cultural meaning of food, capitalizing on positive traditions (respect for bread, the cult of the household, the refusal of waste during fasting periods), and transforming them into modern educational resources.

Thus, the development of an ethical food culture in Romania implies a transition from moralizing discourse to participatory responsibility. Public communication campaigns must go beyond punitive messages (“don’t waste”) and stimulate belonging to a common ideal: that of being part of a fair, sustainable, and solidary food system.

Behavioural and cultural analysis shows that reducing food waste and strengthening food security cannot be achieved through economic or legal instruments alone [64]. A profound change in consciousness is needed—a reconnection between ethics, culture, and everyday action. To the extent that each consumer also becomes a food citizen responsible for his or her choices, food ethics ceases to be an abstract concept and instead becomes a social practice capable of regenerating the balance between food, resources, and social justice.

The results of this behavioural and cultural analysis show that food ethics cannot be separated from the everyday practices of consumers. Ethical awareness, food education, and civic belonging to a fair food system constitute the basic elements of modern food citizenship. This type of citizenship transcends the individual dimension and extends to a collective responsibility, which integrates equity, sustainability, and respect for resources into the very definition of food security.

Therefore, to build a sustainable food system based on social justice and respect for resources, the ethical pillar must be understood as a link between human behaviours and the classical architecture of food security. It directly connects availability with accessibility and use with stability, providing moral coherence to these dimensions. In the absence of an ethical foundation, policies, institutions, and laws risk remaining formal, while social practices continue to reproduce inequalities and waste.

### 4.5. Integrative Synthesis—Strengthening Food Security by Integrating the Ethical Pillar

An integrative analysis of the data and the conceptual framework reveals that food ethics tends to become an emerging pillar of food security, complementary to the four established dimensions: availability, accessibility, utilization, and stability. If these four pillars define the functional structure of food systems, then the ethical pillar gives them moral meaning, societal coherence, and long-term orientation. In a world marked by waste, inequalities and ecological degradation, food security cannot be achieved sustainably without an ethical foundation that regulates the relationship between food, resources, and responsibility [8,9].

#### 4.5.1. Food Ethics and Food Availability

The availability pillar refers to the physical existence of food in sufficient quantity for the entire population. In Romania, where food losses and waste exceed 3.4 million tonnes annually, the availability problem does not stem from a lack of production, but rather from the inequity of distribution and irresponsibility of consumption [10].

From an ethical perspective, food availability not only implies the production of food, but also the real right of everyone to be fed. Food waste, in this context, becomes a form of structural injustice—an expression of the moral imbalance between abundance and scarcity [39].

Therefore, reducing losses and redistributing surpluses must be understood not only as technical or economic actions, but as moral obligations towards society and the environment.

As the state of food security and nutrition in Europe and Central Asia shows, recovering lost food could meet the nutritional needs of millions of vulnerable people. In this sense, the ethic of availability implies a collective responsibility to produce, distribute, and consume food without waste [65].

#### 4.5.2. Food Ethics and Access to Food

The second pillar, access, reflects people’s effective ability to obtain safe, nutritious food that meets their needs. In Romania, access is still affected by social, territorial, and economic inequalities [66].

From an ethical perspective, access to food goes beyond the notion of purchasing power and becomes a matter of human dignity. The FAO states that authentic food security only exists when people can choose food in accordance with the values and traditions of their own community [67].

Surplus redistribution programmes and food banks illustrate the transformation of ethical principles into institutional mechanisms of social justice [56,68]. However, as IPES-Food shows, the ethics of access is not reduced to charity, but involves systemic equity, which requires public policies that eliminate the root causes of food insecurity—income precariousness, lack of food education, and inequality of opportunities [69].

#### 4.5.3. Food Ethics and Food Use

The utilization pillar integrates the nutritional, health, and educational dimensions of food security. From an ethical perspective, food utilization represents the moment of individual responsibility in the food chain: the choice, preparation, and consumption of food become acts of everyday morality.

According to European Commission and IPES-Food, food quality and safety must be viewed in close connection with moral values: respect for health, the environment, and the well-being of others [59,70]. In Romania, although ANSVSA ensures sanitary-veterinary control, ethical responsibility regarding how food is produced and how much is wasted remains insufficiently developed [35].

As IPES-Food notes, “producing safe food is not enough if it comes from systems that destroy ecosystems or exploit people” [8]. Food ethics, therefore, broadens the meaning of the use pillar, adding a moral dimension: responsible consumption and respect for resources become essential indicators of sustainable food use.

#### 4.5.4. Food Ethics and Food System Stability

The stability pillar refers to the ability of food systems to provide consistent access to food, regardless of climatic, economic, or political shocks. Stability is currently threatened by climate change, conflict, and market volatility [52].

The ethics of sustainability involve intergenerational responsibility—caring for the future through present actions. The Food Waste Index 2024 highlights that reducing food waste is one of the most effective ways to increase global resilience, as it conserves resources, reduces emissions, and stabilizes supply [52].

For Romania, integrating ethics into climate change adaptation strategies and agricultural policies is a condition for stability. Food ethics thus offers a framework of moral resilience in which solidarity and prudence become tools for managing food crises.

#### 4.5.5. Integrative Vision

The interconnection of these four pillars confirms the need for a fifth—the ethical pillar—that unifies them into a coherent and sustainable system. If availability ensures the existence of food, access guarantees equity, use reflects responsibility, and stability expresses sustainability, then food ethics provides the moral foundation that makes them all possible.

As FAO and IPES-Food point out, without a solid ethical foundation, food security remains vulnerable to inequalities, waste, and ecological degradation [9,69]. By integrating the ethical pillar, Romania can become a regional model of values-based food governance, in which waste reduction, equity, and solidarity are not simple recommendations, but rather founding principles of food security.

### 4.6. Conceptual Model—Ethical Transition Frameworks Linking Food Ethics, Food Waste, and Food Security

**Figure 4:**
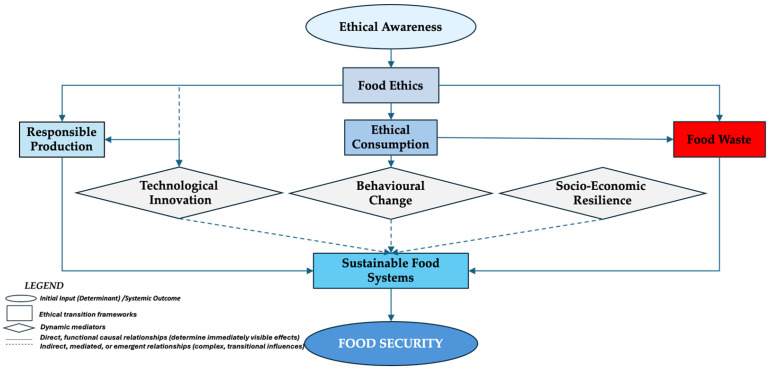
Proposed conceptual model illustrating the ethical transition frameworks linking food ethics, food waste, and food security. Source: Original by authors.

To synthesize the interrelations identified in this study, Figure 4 presents the conceptual model developed by the authors, illustrating how the ethical dimension acts transversally upon the entire food system. The model integrates moral awareness, ethical behaviour, technological innovation, and socio-economic resilience into a coherent framework that links food ethics, food waste, and food security.

The proposed conceptual model presented in Figure 4 describes how the ethical dimension influences production, consumption, and resource management behaviours, ultimately shaping the level of food security. Ethical awareness represents the moral starting point that generates food ethics, which, in turn, guides responsible production and ethical consumption, both of which act to reduce food waste. The mediating socio-technical factors—technological innovation, behavioural change, and socio-economic resilience—facilitate the transition to sustainable food systems, which, in turn, support food security. Thus, food security not only appears as a functional state, but also as the moral outcome of an ethically structured food system.

In this model, solid arrows represent direct relationships (as specified in the conceptual model), while dotted arrows indicate mediated or emergent relationships. This is a theoretical model requiring future empirical validation. Arrow directions represent hypothesized relationships, not validated causal pathways. It is thus suggested that food ethics is not an isolated conceptual component, but rather a foundational ethical dimension that can shape the sustainability and moral quality of the entire food architecture. By integrating moral awareness, responsible production, ethical consumption, innovation, and resilience, the model aligns with the UN Agenda 2030 and the SDGs (SDG 1, SDG 2, SDG 12, and SDG 16).

## 5. Discussion

### 5.1. Reconceptualizing Food Security Through the Ethical Lens

The results obtained in the analysis applied to Romania suggest a broader tension: without an explicit ethical dimension, food security may remain conceptually incomplete. This interpretation aligns with the international evolution of the concept of food security, which, since the FAO definition in 1996, has migrated from a vision focused on availability to one oriented towards food justice, sustainability, and equity [9,71].

This perspective reflects Amartya Sen’s view that hunger and food insecurity are not only effects of lack of resources, but also of inequitable distribution and lack of food rights (“entitlements”), which introduces an ethical foundation into the analysis of food security [65]. In this sense, the proposed ethical pillar extends the entitlements lens beyond distribution alone by bringing moral responsibility for consumption choices and food waste into the analytical frame.

Thus, the ethical pillar proposed in this paper can be read not as a purely local extension, but as a normative component within a broader paradigm in transition. In the vision of FAO and IPES-Food, the transformation of food systems not only requires technological innovation, but also ethical literacy—the capacity of societies to understand and evaluate the moral implications of their own food choices [9,69]. Romania serves here as a representative case study, an example of how traditional values, civic initiatives, and public policies can be integrated into an ethical framework of food governance.

Therefore, redefining food security through the lens of ethics is not just a matter of theory, but a relevant step towards achieving the SDGs, especially SDG 1, SDG 2, SDG 12, and SDG 16, which link access to food to social justice, responsible consumption, and strong institutions.

**Figure 5:**
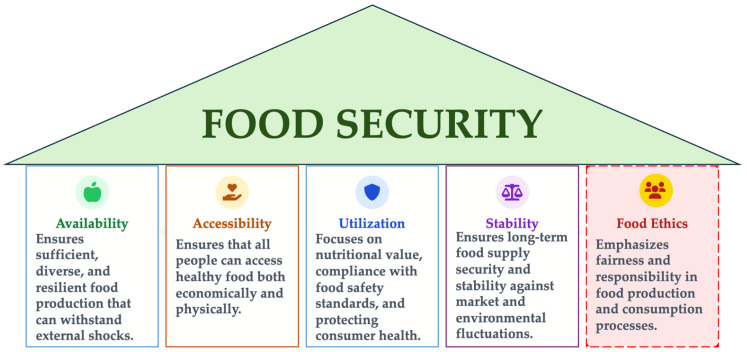
Proposed five pillars of food security, including the food ethics pillar (extension of FAO 1996 framework). Source: Original by authors.

Building on this conceptual analysis, we propose food ethics to be recognized as a distinct, emerging fifth pillar of food security, complementary to the FAO 1996 framework. Figure 5 synthesizes this extension by positioning food ethics alongside availability, accessibility, utilization, and stability.

This model extends the classic framework proposed in 1996, by FAO—built on availability, accessibility, utilization, and stability—by adding an ethical pillar (food ethics), which provides moral coherence and unifying meaning to the entire food system.

Each pillar contributes to the sustainable and equitable functioning of food systems:

- *Availability* refers to sufficient, diversified, and resilient production, capable of withstanding external shocks;
- *Accessibility* refers to equitable and economic access to safe and healthy food for all;
- *Utilization* focuses on nutritional value, food safety, and protecting consumer health;
- *Stability* aims at the long-term security of food supply in the face of market fluctuations and climate risks;
- *food ethics* adds the moral dimension, which links all other components through equity, responsibility, and social justice.

In this way, the conceptual architecture of food security transforms from a strictly functional model to an ethical model in which the moral dimension becomes the foundation that can support sustainability, inclusion, and solidarity in contemporary food systems.

### 5.2. Ethical Food Governance—From Compliance to Moral Responsibility

The legislative and institutional results in Romania suggest a trend of moving from formal compliance to assuming moral responsibility [12,31,52]. Ethical food governance involves a paradigm shift: not just developing rules, but forming collective consciousness around food values.

This perspective aligns with the ideas formulated before, which state that modern food policies must overcome sectoral logic and integrate health, environment, and social equity into a single framework of action [58,63,72,73]. In the Romanian model, the elements of food ethics are implicitly found in the principles of solidarity, responsibility, and sustainability promoted by the UN Agenda 2030. However, what is missing is the institutionalization of this dimension—the creation of a consultative and deliberative framework like the Food ethics Council in the UK or the Dutch Council on Animal Affairs.

At the same time, embedding an ethics-oriented governance layer may face practical challenges: competing economic interests across the food chain, limited administrative capacity for coordination and reporting, uneven logistics for redistribution, cultural resistance to changing long-established consumption norms, and the difficulty of translating ethical principles into measurable indicators. Acknowledging these barriers is important to avoid treating ethical governance as a purely normative aspiration and to clarify the implementation conditions. In this sense, the feasibility of ethics-oriented governance depends on basic enabling capacities, including data availability, interoperable reporting systems, and minimum redistribution logistics.

Such structures ensure coherence between economic objectives and moral principles of food systems, providing governments with ethical guidance in decision-making. In this context, Romania—like other Central and Eastern European countries—may serve as a testing ground for integrating ethics into food governance: through transparency, dialogue between actors, and the inclusion of ethical criteria in the evaluation of public policies. This transition from compliance to moral conscience is, in fact, the essence of the real implementation of SDG 1 (No Poverty), SDG 12 (Responsible Consumption and Production), and SDG 16 (Peace, Justice, and Strong Institutions), which require transparency, fairness, and civic participation.

#### Challenges Around Implementing Ethical Food Governance

Despite the growing recognition of ethical considerations in food governance, the institutionalization of food ethics faces several structural and societal challenges. A first major obstacle relates to economic interests and market pressures. In highly competitive agri-food systems, profit maximization, cost minimization, and supply chain efficiency often take precedence over ethical objectives such as waste reduction, fair access, or responsible production. As a result, economic operators may perceive ethical governance requirements as additional regulatory burdens rather than long-term investments in sustainability [7,8,34].

A second challenge relates to cultural norms and consumer practices. Food-related behaviours are deeply rooted in cultural traditions, social norms, and daily routines. In many contexts, including Romania, food abundance is socially associated with hospitality and well-being, while food waste remains poorly moralized at the household level, especially in urban areas. This cultural framing may limit societal acceptance of ethical governance measures unless they are accompanied by sustained education and awareness-raising efforts [13,39].

Difficulties in measurement and operationalization represent an additional barrier. Unlike traditional indicators of food security, ethical dimensions, such as moral responsibility, food citizenship, or equity in consumption, are inherently complex and multidimensional. The absence of standardized indicators and validated measurement tools can complicate both the monitoring and evaluation of policies and may reduce the willingness of institutions to formally integrate ethical criteria into governance frameworks.

Finally, institutional fragmentation and constraints on governance capacity may hinder implementation. Ethical food governance requires coordination across multiple policy areas—agriculture, social protection, education, environment, and public health—which are often governed by separate institutional logics and mandates. Without clear institutional accountability and cross-sectoral coordination mechanisms, ethical principles risk remaining declarative rather than operational [8,34]. Addressing these challenges not only requires regulatory instruments, but also participatory governance approaches, stakeholder engagement, and the gradual development of measurable ethical indicators. In this sense, ethical food governance should be understood as a progressive process of institutional learning, rather than as an immediately applicable policy model.

### 5.3. From Consumers to Food Citizens—Social Change Through Ethical Awareness

Our proposed ethical pillar should not be interpreted solely as synonymous with preventing food waste. While waste is a highly visible moral signal of irresponsibility towards resources, food ethics also includes broader everyday choices and practices within the food system, such as the quality of diets, equity in access, transparency, and respect for the people and ecosystems involved in production. At the same time, ethical behaviour does not occur in a vacuum: consumers’ “choices” are strongly shaped by the food environment created by commercial practices, marketing, pricing structures, and political incentives. Recognizing these structural constraints helps to avoid an overly moralistic narrative and clarifies that ethical food governance requires both individual action and systemic conditions that enable responsible action.

This broader perspective also intersects with ongoing ethical debates on food transitions, including the difficulty of reducing consumption with a high environmental footprint based on animal products. This study does not treat “ethical consumption” as a single behavioural pathway, but recognizes that shifting to more plant-based diets, where culturally and nutritionally appropriate, is frequently discussed as part of sustainable food system transformation. In Romania, where animal-based preferences remain strong, the ethical pillar therefore not only includes food waste-related routines, but also the gradual realignment of consumption norms with sustainability and equity goals, while avoiding prescriptive statements that go beyond the scope of the current evidence set.

Behavioural analysis has shown that sustainable change in food systems cannot be achieved without the active involvement of consumers. The concept of food citizenship proposes a reconnection between individual rights and responsibilities, between the freedom to consume, and the duty to protect common resources. For example, a “consumer” may treat food primarily as a private commodity: purchasing in excess, discarding edible items close to the “best before” date, and externalizing the consequences. By contrast, a “food citizen” treats food as a shared-value resource: planning purchases, using storage and portioning practices to prevent waste, sharing surplus through family/community networks or food banks, and considering equity and environmental impacts when making everyday food choices.

This idea resonates with the human capabilities approach proposed by Martha Nussbaum, which states that authentic freedom consists of the ability to act in accordance with moral values and to contribute to the common good [74].

Complementary evidence from Romania also suggests that ethical considerations are not only reflected in waste-related behaviours, but also in broader food choices. In this sense, the study conducted by Balan et al. on a sample of 1053 respondents provides an empirical picture of how consumption behaviours in Romania reflect an ethical imbalance between needs, resources, and the right of others to food. The results highlighted a pronounced preference for animal products, especially pork (over 70% of respondents consume more than the nutritional recommendations), along with a low level of consumption of fruits, vegetables, fish, and nuts. This dietary pattern—rich in calories but poor in diversity—has direct consequences on both the health of the population (over half of adults being overweight or obese) and on the sustainability of natural resources and food equity [41].

From the perspective of food ethics, these results confirm that the absence of a moral conscience of consumption amplifies the vulnerabilities of each pillar of food security:

• Affects availability through the overexploitation of resources;
• Distorts access by polarizing between abundance and poverty;
• Limits use through unbalanced diets and food waste;
• Undermines stability through inequalities and pressures on the environment.

At the same time, civic Romanian initiatives demonstrate that ethical values can be translated into concrete social action—in education, donations, and circular economy. These experiences confirm what FAO calls “the moralization of food systems”: the transformation of food from a simple consumer good into a common good managed through solidarity and responsibility [70].

In Romania, the transition from consumer to food citizen is still at its beginning, but the trend is visible, especially in urban areas and among young people. This transformation is not limited to eating habits; it involves the formation of a collective ethical conscience in which everyone understands the impact of their own decisions on others and on the environment.

Food ethics education thus becomes an essential link between knowledge and action and between the educational system and public policies. Therefore, the transition from consumer to food citizen is a fundamental condition for achieving the SDGs—in particular SDG 1 (No Poverty), SDG 2 (Zero Hunger), SDG 12 (Responsible Consumption and Production), and SDG 3 (Good Health and Well-being)—because it transforms food from an individual act into a moral and community commitment.

### 5.4. Policy Implications and Regional Relevance

Although the analysis was conducted on the case of Romania, its implications are regional and global. The countries of Central and Eastern Europe share a common history of economic transition, a traditional food culture and similar challenges regarding food waste and access [28,29]. The proposed ethical model may inform discussions beyond Romania, including at European Union level as a mechanism for strengthening regional food resilience, in full accordance with the UN Agenda 2030 and the European Green Deal.

To make the ethical pillar operational and policy-relevant, several actionable measures could be considered at the national level (with potential regional transferability across Central and Eastern Europe):

• Establish a National Food Ethics Council as an advisory and deliberative body bringing together public authorities, academia, civil society, food businesses, and consumer representatives with a mandate to review major food-related policies through an ethics lens and issue periodic guidance.
• Integrate food ethics education into school curricula with age-appropriate content across primary (grades 1–4), lower secondary (grades 5–8), and upper secondary (grades 9–12), linking food waste prevention, responsible consumption, and social solidarity to everyday practices.
• Develop a Food Ethics Index with measurable indicators (e.g., ethical awareness, self-reported and/or monitored waste-related behaviours, donation practices, understanding of date marking, and support for redistributive mechanisms) to enable monitoring over time and policy benchmarking.
• Implement ethical certification schemes for food businesses (retail, restaurants, catering), encouraging verifiable practices such as prevention plans, donation partnerships, staff training, transparent data marking, and reporting of surplus redirection.
• Introduce targeted fiscal measures to encourage food donation, such as tax deductions or other incentives aligned with national and EU rules, coupled with clear operational guidance to reduce administrative barriers for donors and recipient organizations.

Thus, the ethical pillar does not duplicate the FAO architecture, but complements it, providing moral direction to all its components. This approach resonates with the principles enunciated in the Sustainable Development Report 2024 and with the vision of the UN Agenda 2030, which underlines the need for a value transformation of sustainability policies [75,76].

### 5.5. Towards the Operationalization of the Food Ethics Pillar

While the food ethics pillar is introduced in this study primarily as a conceptual and normative extension of the food security framework, its relevance for research and policy depends on the possibility of future operationalization. To address this aspect, Table 9 proposes a set of illustrative proxy indicators that could support the empirical measurement and monitoring of the food ethics pillar. These indicators are not validated and are not empirically applied in the present analysis; rather, they are intended to provide a structured conceptual starting point for future research, comparative assessments, and policy-oriented evaluation (Table 9).

### 5.6. Limitations and Future Research

This research is based on secondary data (FAO, Eurostat, Eurobarometer, MADR) and is therefore limited by its availability and granularity. The lack of recent national surveys on the ethical perception of food consumption in Romania was partially compensated by using indirect sources (European surveys, studies published in the literature). In the future, it would be useful to extend the research through a qualitative focus group study or by applying a questionnaire to a national sample. In the same sense, as the analysis is based on a single-country case study, the findings are context-dependent and may not be directly generalizable to other national settings. However, the conceptual framework and the identified food waste–food insecurity dynamics offer transferable insights for countries facing similar governance and behavioural challenges.

In addition, the statistical indicators used in this study refer to the period 2020–2022, which represents the entire set of data reported by Romania from the beginning until the time of analysis. This temporal limitation means that the study cannot capture any developments that occurred after this period. This period also overlaps with the COVID-19 pandemic, which may have temporarily influenced food-related behaviours, particularly at the household level, further limiting the generalizability of the observed patterns beyond crisis conditions. Although a data series was manually constructed in Excel for exploratory purposes, to ensure continuity and comparability of the indicators, the use of aggregated secondary data instead of primary data collection may introduce certain limitations in terms of accuracy and level of interpretative detail. Future research could integrate microdata or household-level monitoring tools to more accurately capture regional and local behavioural variations.

Future research directions could include

• Comparative studies between EU states on the integration of ethics into food policies;
• Assessing ethical perceptions among economic actors in the agri-food chain;
• Analysis of the impact of sustainability education on consumer behaviour.

Such studies would not only strengthen the theoretical foundation of the ethical pillar, but also the capacity to implement the UN Agenda 2030, contributing to the simultaneous achievement of SDG 1, SDG 2, SDG 12, and SDG 13.

Therefore, integrating ethics into food security emerges as a promising direction for future theoretical and applied research, rather than merely a conceptual option. The results obtained from the applicative analysis for Romania, but relevant on an international scale, show that the ethical pillar may help reframe food security into a common social and moral project, aligned with the principles of the UN Agenda 2030.

Considering these findings, future research should focus on the empirical validation and measurement of the proposed food ethics pillar, including the testing and refinement of the illustrative proxy indicators outlined in this study. Thus, future conceptual work could contribute to expanding the contemporary understanding of food security should be enlarged from a functional architecture to an axiological one in which food is no longer just an economic good, but also a moral and community good. In this framework, the introduction of the ethical pillar does not contradict the FAO definition, but rather complements and humanizes it, transforming food security from a technical objective into a global ethical commitment, essential for achieving the SDGs (SDG 1, SDG 2, SDG 12, SDG 13, and SDG 16).

## 6. Conclusions

This study suggests that food ethics is not a secondary dimension, but rather a potentially fundamental normative dimension of food security. The case of Romania illustrates how social values, public policies, and civic engagement can converge in an approach to food governance based on moral responsibility. By linking responsibility to rights and justice to sustainability, food ethics redefines food security as a holistic system in which production, distribution, and consumption are united under the same moral framework [8,9].

The four established pillars of food security—*availability, accessibility, utilization, and stability*—respond to each functional dimension of the food system. However, their long-term adequacy may be constrained without ethical coherence that guarantees equity, inclusion, and respect for life. Integrating the ethical dimension into each of these pillars transforms food systems from efficiency mechanisms into instruments of justice. At the same time, by virtue of its scope and normative force, food ethics is proposed for conceptual recognition as an emerging fifth pillar intended to provide moral structure and coherence to the entire conceptual architecture [70,77].

The implications of this approach may extend beyond Romania’s borders. In regions undergoing economic or social transition, such as Central and Eastern Europe, ethical food governance could serve as a stabilizing force, contributing to aligning public policies with the principles of the UN Agenda 2030. Integrating food ethics into national strategies directly supports the achievement of SDG 1 (No Poverty), SDG 2 (Zero Hunger), SDG 12 (Responsible Consumption and Production), SDG 13 (Climate Action), and SDG 16 (Peace, Justice, and Effective Institutions) [76].

At the same time, this study highlights the need for future research aimed at operationalizing food ethics through measurable indicators—such as a Food Ethics Index—and exploring validation pathways through:

(i) Dedicated survey modules on ethical awareness and food-citizenship practices;
(ii) Behavioural proxies and, where feasible, small-scale observational approaches to food waste-related routines;
(iii) Longitudinal or cross-country comparisons once longer time series become available.

Ethical and food literacy, promoted through education and civic participation, is an essential condition for transforming moral principles into concrete public policy outcomes [9,60].

In essence, food ethics transforms food security from a technical objective into a moral mission. At the same time, this should be read as a conceptual proposition: the ethical pillar is proposed here as a distinct, emerging fifth pillar alongside the four FAO pillars with its own normative content and analytical role. It calls for a redefinition of human responsibility for food not just as a resource, but rather as a relationship that unites individuals, communities, and the planet. In a world of profound contrasts—between the global North and the global South, but also between neighbourhoods within the same city—food ethics may serve as a shared consciousness of justice and solidarity. It reminds us that waste and scarcity and abundance and hunger can coexist within the same borders and that true sustainability is not measured by production alone, but also by equity. By advancing food ethics as a pillar of food security, this study argues that the future of food systems not only depends on our ability to produce enough, but also on the moral maturity to share fairly, respect resources, and recognize the interdependencies that connect us between people, regions, and continents. Future research should operationalize and validate this pillar through measurable indicators (e.g., a Food Ethics Index), dedicated survey modules on ethical awareness and food citizenship practices, and longitudinal or cross-country comparisons as longer time series become available.

The true measure of food security is not just about “having something to eat”, but rather about ensuring that no one goes hungry while someone else can live in abundance.

## Figures and Tables

**Table 1:** Food waste in Romania by sector in tonnes [39].

Year	Total	Primary Production of Food—Agriculture, Fishing and Aquaculture	Manufacture of Food Products and Beverages	Retail and Other Distribution of Food	Restaurants and Food Services	Total Activities by Households
2020	3,201,048	699,920	316,507	39,787	485,827	1,659,007
2021	3,392,056	699,920	268,349	36,510	589,365	1,797,912
2022	3,452,143	613,337	375,577	42,864	543,244	1,877,121

**Table 2:** Food waste in Romania by sector in kg/capita [39].

Year	Total	Primary Production of Food—Agriculture, Fishing and Aquaculture	Manufacture of Food Products and Beverages	Retail and Other Distribution of Food	Restaurants and Food Services	Total Activities by Households
2020	166	36	16	2	25	86
2021	177	37	14	2	31	94
2022	181	32	20	2	29	99

**Table 3:** Percentage contribution of food waste from food chain segments to the variation in total waste *C_i_* in %.

Segment	C_i_ (2021–2020) (%)	C_i_ (2022–2021) (%)
PPF	0.00	26.60
MPF	16.39	32.94
RDF	1.12	1.95
RFS	35.23	14.17
TAH	47.27	24.33

**Table 4:** The values of the correlation coefficients *r* and the determination coefficients R^2^ between the annual series of each food chain segment and the annual series of total waste, r and R^2^, are dimensionless.

Segment	r	R^2^
PPF	−0.69	0.47
MPF	0.29	0.08
RDF	0.21	0.04
RFS	0.77	0.59
TAH	0.99	0.98

**Table 5:** The values of the regression coefficients β and the coefficients of determination R^2^ used to analyze the interactions of food waste between food chain segments, considering RFS as the causal variable—β expressed as outcome units per 1 unit of predictor; R^2^ as dimensionless.

Predictor	Outcome	β	R^2^
RFS	PPF	−1.079	0.621
RFS	MPF	0.604	0.343
RFS	TAH	3.100	0.836
RFS	RDF	0.029	0.275

**Table 6:** Residual covariance values used to describe interactions between economic sectors regarding food waste, considering RFS as the causal variable—residual covariances are dimensionless (as estimated by JASP SEM).

Interaction	Residual Covariances
PPF-MFP	−1.300 × 10^−8^
PPF-TAH	−8.622 × 10^−9^
PPF-RDF	−2.370 × 10^−7^
MFP-TAH	8.937 × 10^−9^
MFP-RDF	2.274 × 10^−7^
TAH-RDF	1.613 × 10^−7^

**Table 7:** The values of the regression coefficients β and the coefficients of determination R^2^ used to analyze the interactions of food waste between food chain segments, considering TAH as the causal variable—β expressed as outcome units per 1 unit of predictor; R^2^ as dimensionless.

Predictor	Outcome	Β	R^2^
TAH	PPF	−0.392	0.941
TAH	RFS	0.270	0.836
TAH	MPF	0.263	0.746
TAH	RDF	0.014	0.680

**Table 8:** Residual covariance values used to describe interactions between economic sectors regarding food waste, considering TAH as the causal variable—residual covariances dimensionless (as estimated by JASP SEM).

Interaction	Residual Covariances
PPF-RFS	2.082 × 10^−7^
PPF-MFP	−1.309 × 10^−7^
PPF-RDF	−2.199 × 10^−6^
RFS-MFP	−1.039 × 10^−7^
RFS-RDF	−1.746 × 10^−6^
MFP-RDF	1.274 × 10^−6^

**Table 9:** Illustrative proxy indicators for operationalizing the proposed food ethics pillar—conceptual proposal *.

Food Ethics Dimension	Illustrative Proxy Indicator	Unit/Scale (Measurement)	Potential Data Source/Method	Rationale (Link to Pillar)
Ethical awareness in food decisions	Share of population reporting that ethical considerations influence food choices	% (0–100) of respondents	Population survey (national or targeted)	Captures explicit moral salience in consumption decisions
Responsibility and agency	Intention–action gap for food waste prevention (difference between stated intention and reported behaviour)	Score difference (e.g., Likert 1–5 intention minus Likert 1–5 behaviour) or % gap	Survey module combining intentions + self-reported practices	Quantifies the “values–actions” divergence central to food ethics
Prosocial redistribution (solidarity)	Frequency of food donation/redistribution practices (households)	times/month or times/year; alternatively, the % donating at least once/year	Household survey; administrative data from food banks (where available)	Reflects ethical commitment to avoiding surplus waste and supporting access
Avoidance of edible food disposal	Self-reported edible food discard frequency	times/week or times/month; or % reporting discard in last 7 days	Household survey (behavioural frequency items)	Directly links ethical restraint/household norms to waste prevention
Food citizenship participation	Participation in food-related civic/educational initiatives (workshops, campaigns, community actions)	% participating (yes/no) and/or count/year	Survey; program attendance records	Captures civic engagement and norm diffusion around responsible consumption
Ethical literacy (knowledge)	Awareness of food waste impacts and prevention practices (knowledge score)	Index score (0–10, 0–100)	Survey-based quiz/knowledge battery	Indicates the capacity to act ethically (knowledge as enabling factor)
Norms and moral discomfort	Strength of moral norms against wasting food (agreement with norm statements)	Likert score (1–5 or 1–7) or composite index	Survey (validated norm items can be used later)	Measures of internalized ethical norms that predict behaviour
Institutional support environment	Exposure to/availability of redistribution and prevention infrastructure (e.g., food donation channels)	Binary (0/1) or count of available options	Local administrative mapping; survey	Context indicators enabling ethical action (structural precondition)

* These proxy indicators are proposed for conceptual operationalization and future empirical research. They are not validated measures and are not applied in the present analysis.

## Data Availability

The original contributions presented in this study are included in the article material. Further inquiries can be directed to the corresponding authors.
